# Falling in Love With Work: The Effect of Enterprise Social Media on Thriving at Work

**DOI:** 10.3389/fpsyg.2021.769054

**Published:** 2021-11-15

**Authors:** DongXu Liu, Beigang Hou, Yuanyuan Liu, Pingqing Liu

**Affiliations:** ^1^School of Management and Economics, Beijing Institute of Technology, Beijing, China; ^2^Advisory Administration, Korn Ferry, Beijing, China

**Keywords:** enterprise social media, thriving at work, challenge stressors, obstructive stressors, the social embeddedness model

## Abstract

Using a survey of 300 employees in different types of enterprises and different positions, this study verified that the use of enterprise social media has a positive effect on employees’ work exuberance. The study separately examined the effects of social media applications for work tasks and social tasks. Both types of applications had a positive impact on employees’ work exuberance. The study also identified the mediating role of challenge and obstructive stressors in this relationship. Work-related social media applications enhanced employees’ exuberance by reducing obstructive stressors, and social-related social media applications enhanced employees’ exuberance by reducing challenge stressors. The implications of these findings are that managers should pay attention to the use of enterprise social media, especially for social tasks, as this can enhance employees’ sense of exuberance.

## Introduction

More and more enterprises are using social media. Between 2020 and 2021, more than 30 million enterprises used social media for the first time. Social media platforms in work contexts have both social and work applications (Luqman et al., 2021). Studies have shown that social media can expand people’s social relations and networks and reduce social tension; however, it may also increase anxiety and impair people’s rational thinking (Cheng et al., 2021). Therefore, the impact on employees of enterprise social media platforms is unclear. Will social media make us love our work more?

Enterprise social media has created new behavior patterns. Social media technology can further improve the efficiency of information transmission within an enterprise and promote communication between management and frontline employees. The use of social media breaks the bottlenecks in information dissemination that frequently occurs in offline offices and makes it possible to spread organizational decisions to the grassroots level quickly, which helps enterprises create an open and transparent organizational atmosphere, break through the internal barriers that characterize traditional organizations, share resources, open processes, reduce communication costs, enhance organizational vitality, and influence turnover intentions (Murad et al., 2020). Within an organization, transparent resources and information provide employees with multiple channels to share knowledge and more opportunities to learn and grow (Paul et al., 2013). As the use of enterprise social media becomes more common, its impact on employees has gradually become of interest to scholars. Some studies have examined the different effects of using social media for work tasks and for social tasks (Patroni, 2016). Their results have shown that social media can improve employees’ organizational commitment, but using social media for work tasks mainly affects normative commitment, whereas its use for social tasks mainly affects emotional commitment. Studies (e.g., Eliane et al., 2013) of the impact of enterprise social media on employee job satisfaction have found that work-related social media has no significant impact on employee job satisfaction, but social-related social media has a favorable impact on job satisfaction.

Scholars have begun to pay more attention to the concept of thriving at work, which measures two dimensions of employees’ enjoyment of work: maintaining vitality at work and learning (Demerouti and Bakker, 2011). Thriving at work is not only an important manifestation of individuals’ sustainable development but is also of great importance for organizational performance and for the overall well-being of society (Sigala and Chalkiti, 2015). Therefore, there have been many studies of the personal and organizational strategies that enhance individuals’ sense of exuberance. According to the social embeddedness model, work resources and work situations affect individuals’ initiative behavior, which in turn shapes their experience of thriving at work (Lesener et al., 2018). The use of enterprise social media enhances internal information sharing, creating an atmosphere of mutual trust and respect. If this changes employees’ access to knowledge, relationships, and other resources at work, the employees’ sense of thriving at work will also change (Tucker and Goodings, 2017). However, there have been very few theoretical and empirical studies of the mechanism through which enterprise social media affects thriving at work. In particular, there have been no studies of how stress affects the relationship between social media use and thriving at work. Only a few studies (e.g., Yu et al., 2018) have considered the impact of social media use on employees’ stress or on employees’ work status.

Therefore, whether the use of social media in enterprises promotes employees’ exuberance remains an empirical question. Can the use of social media make employees love their work more? This paper studied this problem from the perspective of stress.

## Theoretical Background

### Enterprise Social Media

The use of social media in enterprises allows employees to release, transmit, search, and receive information through social media platforms. As a tool for the internal management of enterprises’ needs, enterprise social media functions somewhat differently than non-enterprise social media. Within enterprises, social media is mainly used for communication among employees or to a specific employee. Users can edit and revise documents with colleagues and everyone can view the posted information and files (Lepine et al., 2004).

Paul et al. (2013) divided enterprise social media into three types based on common social media tools. The first type is public social media, which includes platforms such as WeChat and QQ. For enterprises, public social media platforms have become channels for external publicity and after-sales service for external customers. As such platforms also have non-work social functions for employees (Permatasari et al., 2013), their application in work settings will have an impact on employee behavior. The use of public platforms can promote information transfer among employees and enhance mutual understanding (Parnin and Garg, 2007). However, it is also accompanied by problems, such as blurring the boundary between work and home life, privacy leakage, and extension of work into non-working hours, among others. The second type of enterprise social media is private platforms, which may operate with either commercial proprietary software or open-source software, but are only for internal employees’ use (Kuegler et al., 2015). Private platforms usually have a range of functions, including dynamic sharing, chat, and document sharing. The third type is social media tools independently developed by an enterprise’s internal technical team to meet the specific needs of the enterprise (Kwon and Wen, 2010). In general, these tools are not limited to communication functions and provide various functions based on the actual work needs of employees, such as live broadcasting, online document editing, announcement releases, and so on.

Boyd and Ellison (2010) developed an alternative two-dimensional classification of enterprise social media: those with social applications and those with work applications. Work-related social media tasks focus on workflow, work cooperation among employees, work plan arrangement, and work progress follow-ups, all of which can promote the integration and innovation of organizations’ internal and external resources. The social applications of enterprise social media involve tasks that contribute to a harmonious working atmosphere in an organization (Hayes and Carr, 2021), help managers better understand the mood and state of employees, provide timely organizational support, and cultivate team cohesion.

Studies of the work attributes of enterprise social media have focused on the increased efficiency of information transmission associated with the use of social media, and the resulting improvements in an open and transparent organizational atmosphere (Paul et al., 2013), employee knowledge management and sharing, employee performance, enterprise communication, and relationship management.

Enterprise social media can act as a “social lubricant” simplifying connections and communication (Saeed and Ilkhanizadeh, 2021). When employees share private information on this type of media, it creates a harmonious organizational atmosphere and cultivates a sense of belonging among new employees. Enterprise social media can help the growth of social capital within the organization. The social activities of colleagues become resources for establishing connections with unfamiliar colleagues and making more informal contacts. Such media can also make it easier to adapt to the working environment and increase employees’ feelings of psychological safety, which helps introverted or self-effacing people to form relationships (Paul et al., 2013). However, enterprise social media is a double-edged tool. Although a variety of relationships can be established through this type of media, they are generally not strong relationships, and some cannot even be classified as interpersonal relationships (Boyd and Ellison, 2010). In addition, the use of social media for work can blur the boundary between employees’ private and public lives and between private and public relations. The group communication mode of such media also combines social relations of varying strength, which increases complexity of the interpersonal relations.

### Challenge and Obstructive Stressors

Cavanaugh et al. (2000) developed the challenge-obstructive stressor model. A challenge stressor is a stressor that can create challenges but may also lead to positive outcomes (Hase et al., 2018). It can have a positive impact on career development and produce positive work results. A common challenge stressor is time stress, which occurs when the amount of work or its complexity makes finish the work on time a challenge (Hsu and Fan, 2008). An obstructive stressor is a stressor generated by restrictions that hinder personal development goals. Common obstructive stressors include bureaucracy, organizational politics, job insecurity, and career stagnation (Boswell et al., 2004).

Challenge stressors usually have a positive impact, because although they create short-term stress for individuals (Cavanaugh et al., 2000), individuals believe that overcoming the challenge will lead to promotions, salary increases, and other positive returns. Therefore, challenge stressors generally have a positive influence on behavior. In contrast, obstructive stressors usually have a negative impact on employees, as obstructive stress consumes employees’ own resources without any expectation of future returns and benefits. Therefore, obstructive stressors generally promote negative coping strategies, such as resignations and strikes (Hobfoll, 1989).

In this study, challenge and obstructive stressors were not considered mutually exclusive (Hase et al., 2018). According to the theory of cognitive interaction, stress is neither a stable personal trait nor a situational stimulus, but a product of the interaction between situations and individuals’ processing abilities. Thus, in a complex situation, individuals may make different evaluations of challenge and obstructive stressors and may even experience both types of stressors at the same time (Zhang Y. et al., 2015).

### Thriving at Work

Thriving at work is a state in which an individual is motivated to learn and grow at work. Such employees are full of vigor and vitality at work and experience a sense of self-development. It is a positive subjective experience and feeling (Niessen et al., 2012), and it is a temporary psychological state rather than a stable personal characteristic.

There are two dimensions of thriving at work: vitality and learning. Vitality is the positive feeling of being full of energy and learning is the desire to acquire knowledge and skills (Porath et al., 2012). The social embeddedness model is a widely accepted theoretical model for explaining the mechanisms of the formation of thriving at work. According to the social embeddedness model, thriving at work is largely shaped by the characteristics of the work situation and the social support system. Studies Porath et al. (2012) have identified some of the characteristics that affect thriving at work. The departmental characteristics that affect the sense of thriving at work are independent decision-making, extensive information sharing, respect, and trust. The main resources provided by the social support system are positive significance, positive emotional resources, and relationship resources.

When an individual’s department provides an environment that allows for independent decision-making, information sharing, trust, and respect and provides the individual with knowledge, positive significance, positive emotional resources, and relationships, the individual is more likely to engage in active work behaviors, such as concentrating on work, exploring, and establishing better relations within the enterprise. Employees’ proactive work behavior can also promote thriving at work, creating a positive cycle (Xu et al., 2019). As employees’ vitality increases, it promotes learning, which in turn increases employees’ thriving at work. Exuberant employees are more likely to be physically and mentally healthy and are more likely to achieve long-term personal development.

Both personal and organizational factors affect thriving at work (Xu et al., 2019). The personal factors include mental health, physical health, employees’ recognition of work, and self-determination. Porath et al. (2012) identified four organizational factors that promote a pluralistic and inclusive organizational climate as follows: giving employees decision-making power; open and transparent information, especially the communication of the company strategy and other related information; low levels of uncivil behavior within the organization; and feedback on performance.

Porath et al. (2012) argued that social media can act as a social lubricant by increasing the daily contact between employees and thus enhance employees’ sense of belonging to a team. Increasing the contact opportunities between employees may also help employees to thrive at work. Social media can also help employees to obtain organizational information more efficiently and help management to provide timely performance feedback and support, improving employees’ work environment.

## Research Design and Hypothesis Development

### Social Media Usage and Thriving at Work

According to the social embeddedness model, work resources and work situations affect individuals’ active work behavior, which in turn affects individuals’ sense of thriving at work. The use of enterprise social media is conducive to the internal sharing of information and the formation of an atmosphere of mutual trust and mutual respect (Luqman et al., 2021). Such work situations are conducive to the generation of a sense of exuberance. Furthermore, enterprise social media applications provide employees with access to more organizational information and more organizational team members, which is also conducive to the generation of thriving at work.

Enterprise social media allows employees to work online with colleagues; for example, they can write online documents together, make queries, and share relevant information and knowledge. Extensive information and knowledge sharing help to build a more trusting and respectful organizational atmosphere (Sun et al., 2019). This type of media also gives employees opportunities to establish relationships with more people in the organization. Rich relationship resources allow employees to obtain more knowledge resources, which are conducive to individual learning. The use of social media for work tasks can promote information and knowledge sharing (Lepine et al., 2004), accelerate the flow and precipitation of knowledge resources within the organization, and stimulate knowledge innovation. Its use for social tasks can enhance the atmosphere of trust and respect and increase relationship resources. Both types of users have a positive impact on departmental characteristics and individuals’ resource pools. Based on the above discussion, this study made the following hypotheses.

Hypothesis 1(a): The use of enterprise social media for work tasks has a significant positive impact on thriving at work.
Hypothesis 1(b): The use of enterprise social media for social tasks has a significant positive impact on thriving at work.

### Enterprise Social Media Usage and Challenge and Obstructive Stressors

According to the conservation of resources theory, individuals facing a potential or actual loss of resources or having difficulty obtaining returns on resources they have invested in will experience stress (Hobfoll, 1989). Many personal traits such as self-esteem and self-efficacy provide individuals with protection from stressors (Lesener et al., 2018). Social support is also an important resource because social contacts can reduce individuals’ perception of stress, thus helping to preserve and reduce the loss of personal resources.

Both challenge and obstructive stressors consume an individual’s resources, but a challenge stressor generally involves a complex work problem and an urgent deadline. When employees believe that completing the tasks or taking on the heavy workload will bring benefits that will replace the individual resources consumed by the work, they will regard the stressor as a challenge stressor. Obstructive stressors include obstacles such as bureaucratic barriers, cumbersome procedures, or job insecurity. When employees think that the work they are expected to do will consume their resources and give them none in return, they will regard the stressor as an obstructive stressor.

The use of enterprise social media can be regarded as both use of work resources and consumption of work resources and may produce both positive and negative outcomes (Westman et al., 2004). Work-related applications of social media can increase the transparency and fairness of an office environment, as all employees can query all public information in the organization. Knowledge sharing and work coordination can be conducted on social media, which is conducive to reducing bureaucratic procedures and organizational politics, improving work efficiency, and enhancing employees’ sense of work security, thus reducing the obstructive stressors in the workplace. However, its use extends the temporal and spatial boundaries of the office, making it possible to work anytime and anywhere. This means that employees need to invest more energy and resources in their work and the boundaries between personal life and work become blurred, which creates challenging stressors for employees. The use of social media enriches emotional communication between employees and increases the scope of employees’ social interactions. This provides employees with more social support and networking resources, thus reducing both challenges and obstructive stressors. Based on the above insights, the authors made the following hypotheses.

Hypothesis 2(a): The use of enterprise social media for work tasks has a significant positive impact on the strength of challenge stressors.
Hypothesis 2(b): The use of enterprise social media for work tasks has a significant negative impact on the strength of obstructive stressors.

Hypothesis 3(a): The use of enterprise social media for social tasks has a significant negative impact on challenge stressors.
Hypothesis 3(b): The use of enterprise social media for social tasks has a significant negative impact on obstructive stressors.

### Effects of Challenge and Obstructive Stressors on Thriving at Work

According to the social embeddedness model (Spreitzer et al., 2005), the availability of the resources required by individuals to complete their work will affect the employees’ vigor. The resources required to overcome the challenges and obstructive stressors can be considered job requirements (Wang et al., 2020). If individuals have to wait for the resources they spend overcoming stressors to be replaced, they will feel anxiety, depression, anger, and other negative emotions, which will have an adverse impact on their emotional, physical, and mental health. Therefore, by exhausting employees’ resources, challenging and obstructive stressors have a negative impact on their sense of job exuberance.

However, studies have shown that challenging stressors can also have a positive impact on individuals. Podsakoff et al. (2007) found that the two common types of challenge stressors, time limits, and learning demand, can positively affect the learning dimension of thriving at work while negatively affecting the vitality dimension of thriving at work. By definition, to thrive at work employees need both learning opportunities and vitality. Therefore, challenge stressors have a negative impact on thriving at work.

Hypothesis 4(a): Challenge stressors have a significant negative impact on thriving at work.
Hypothesis 4(b): Obstructive stressors have a significant negative impact on thriving at work.

### Mediating Roles of Challenge and Obstructive Stressors

Job requirements such as time limits, task overload, or role conflict are sources of job stress. Job control is regarded as an outlet for job stress, and it generally consists of the ability to make independent decisions at work (Ito and Brotheridge, 2003). Therefore, social support may mediate the relationship between job requirements and job stress.

The use of enterprise social media has changed work environments and the requirements, processes, and level of employees’ decision-making authority. Employees can use social media at work to search for internal information, thus enhancing their control over their work. When employees carry out social activities on this type of media, they can more easily get work support from colleagues and leaders in the organization, which improves their sense of social support. When the job requirements are high but employees have control over the work and strong social support, they will have a more positive attitude toward work. Even when there are many job requirements, they will be happy to improve their work skills through learning and to successfully complete their work tasks, which is the definition of thriving at work. Based on the above insights, the authors made the following hypotheses.

Hypothesis 5(a): Challenge stressors mediate the relationship between the use of enterprise social media for work tasks and thriving at work.
Hypothesis 5(b): Obstructive stressors mediate the relationship between the use of enterprise social media for social tasks and thriving at work.

Hypothesis 6(a): Challenge stressors mediate the relationship between the use of enterprise social media for work tasks and thriving at work.
Hypothesis 6(b): Obstructive stressors mediate the relationship between the use of enterprise social media for social tasks and thriving at work.

## Materials and Methods

### Study Design

**FIGURE 1:**
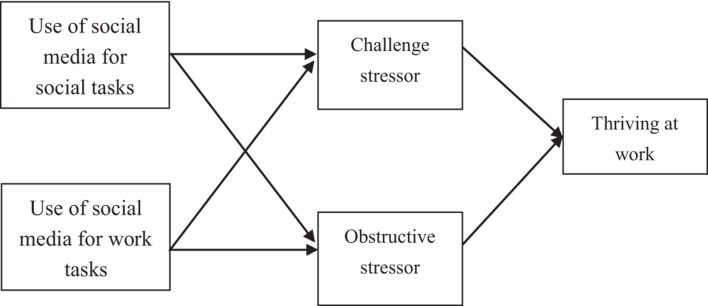
The relationship between social media usage and thriving at work.

This study took work-related and social-related social media use as independent variables, work exuberance as the dependent variable, and challenge or obstructive stressors as the intermediary variable. The model is shown in Figure 1.

Enterprise social media usage was measured using the scale developed by Leidner et al. (2010). It has 13 items, including 8 items for work-related enterprise social media usage and 5 items for social-related enterprise social media usage. Challenge and obstructive stressors were measured using the scale developed by Cavanaugh et al. (2000). It has 11 items, of which 6 items measure challenge stress. Work vitality was measured using the scale developed by Porath et al. (2012). It has 10 items, which are divided into two dimensions: learning and vitality.

The sample collection strategy targeted people with work experience. A paid questionnaire on the Credamo network platform was used to collect sufficient data. Credamo questionnaires offer eight functional settings: release channel, release quantity, quality control, sample feature setting, response sets, conclusion setting, questionnaire compensation, and expense setting, and forwarding reward. These settings can be used to set demographic characteristics and accurately locate a survey population that meets your research needs. Quality control and answer settings can further control the subjects’ answering conditions, further realize quality control, and fully ensure the reliability and effectiveness of the data.

Invalid questionnaires were eliminated, and the following three criteria were used to ensure that the results were not biased by participants not completing the questionnaires carefully: (1) the questionnaire was provided with reverse scoring questions to eliminate answers that are obviously inconsistent; (2) specified option questions were set, and questionnaires with answers that were inconsistent with the specified options were eliminated; and (3) questionnaires with the same answers to 10 consecutive questions were eliminated.

SPSS and Amos software programs were used to analyze and process the results.

### Data Collection

Of the 303 questionnaires distributed on the network platform, 300 were returned and 270 were valid, giving an effective return rate of 90%. Among the participants, 52.2% were men and 47.8% were women. Most of the participants (98.1%) were between 21 and 40 years old. The majority of the participants had high educational achievements: 261 (88.5%) had a Bachelor’s degree or above. In terms of work experience, 0.7% of them had worked for 1–3 years, 43.3% have worked for 4–6 years, 20.7% have worked for 7–10 years, and 9.3% have worked for more than 10 years. The job types were distributed among finance (12.6%), technology (40%), marketing (7.8%), human administration (21.1%), and production (17%).

### Ethics

All of the participants understood the purpose of the study and agreed to participate in the study. The researchers used codes to replace the personal information of specific participants to ensure the security of the participants’ private information.

## Results

Amos 26.0 was used to conduct confirmatory factor analysis on five variables: work-related use of enterprise social media, social-related use of enterprise social media, challenge stressors, obstructive stressors, and thriving at work. The results, presented in Table 1 [^2^/*df* = 1.683, composite fit index (CFI) = 0.906, incremental fit index (IFI) = 0.907, root mean square error of approximation (RMSEA) = 0.049, root mean square residual (RMR) = 0.052], indicated that the five-factor model had a better fit than the other models, with good structural validity among the variables, a simple fit, and an overall goodness of fit. Overall, the results of our confirmatory factors’ analysis showed that the five-factor model, composed of work-related enterprise social media use, social-related enterprise social media use, challenge stressors, obstructive stressors, and thriving at work, had a better fit than any of the other factors’ models.

**TABLE 1:** Confirmatory factor analysis.

	**Model**	** $\frac{x^{2}}{d\,f}$ **	**CFI**	**IFI**	**RMSEA**	**RMR**
**1**	**Five-factor model** (Xa, Xb, Ma, Mb, Y)	1.683	0.906	0.907	0.049	0.052
**2**	**Four-factor model** (Xa + Xb, Ma, Mb, Y)	2.197	0.808	0.811	0.067	0.056
**3**	**Three-factor model** (Xa + Xb, Ma + Mb, Y)	2.624	0.739	0.741	0.078	0.074
**4**	**Two-factor model** (Xa + Xb + Ma + Mb, Y)	3.441	0.606	0.610	0.095	0.095
**5**	**One-factor model** (Xa + Xb + Ma + Mb + Y)	4.529	0.429	0.435	0.115	0.120

*Xa—work-related enterprise social media use; Xb—social-related enterprise social media use; Ma—challenge stressor; Mb—obstructive stressor; and Y—a sense of thriving at work.*

The means, standard deviations, and correlations are presented in Table 2. There were positive correlations between thriving at work and the use of enterprise social media for social tasks (*r* = 0.548, *p* < 0.01), thriving at work and the use of enterprise social media for work tasks (*r* = 0.285, *p* < 0.01), the use of enterprise social media for work tasks and obstructive stressors (*r* = −0.14, *p* < 0.05), and the use of social media for social tasks and challenge stressors (*r* = −0.152, *p* < 0.05). Thriving at work was negatively correlated with challenge stressors and obstructive stressors.

**TABLE 2:** Descriptive statistical analysis and correlations (*N* = 270).

**Variable**	***M* (*N*)**	***SD***	**1**	**2**	**3**	**4**	**5**	**6**	**7**	**8**	**9**	**10**	**11**	**12**
Sex	1.48	0.5	1											
Age	2.4	0.53	0.08	1										
Education	3.09	0.39	0.06	0.03	1									
Work experience	4.12	0.93	0.11	0.649**	0.01	1								
Company type	2.8	1.46	0.1	0.01	−0.121*	−0.05	1							
Company size	2.61	0.96	0.06	−0.04	0.11	0.08	−0.195**	1						
Position	2.36	1.39	0.273**	0.02	−0.01	−0.04	0.1	−0.156*	1					
Xa	4.35	0.33	−0.06	−0.01	0.02	0.05	0.07	−0.04	0.03	1				
Xb	3.91	0.7	0.04	0.06	−0.02	0.04	−0.02	0.145*	−0.04	0.293**	1			
Ma	3.35	0.84	0.05	−0.11	−0.02	−0.142*	−0.06	−0.05	−0.01	−0.04	−0.152*	1		
Mb	2.62	0.85	0.01	−0.07	−0.05	−0.222**	0	−0.150*	−0.01	−0.140*	−0.11	0.500**	1	
Y	4.32	0.46	−0.179**	0.06	0.04	0.165**	−0.03	0.09	−0.11	0.548**	0.285**	−0.194**	−0.371**	1

Xa, work-related enterprise social media use; Xb, social-related enterprise social media use; Ma, challenge stressor; Mb, obstructive stressor; Y, a sense of thriving at work. *correlation is significant at the 0.05 level, **correlation is significant at the 0.01 level.

As shown in Table 3, the bootstrap analysis also showed that there were some mediators between work-related enterprise social media use and job exuberance, with the direct effect accounting for 93.87% and the indirect effect accounting for 6.13% of the outcomes. The mediating effect of challenge stressors on the relationship between work-related enterprise social media use and job exuberance was not significant, and there was no side-by-side mediating effect of challenge or obstructive stressors. Therefore, H5a was not supported, but H5b was supported. As shown in Table 4, when the use of enterprise social media for social tasks was the independent variable, the bootstrap analysis showed that challenge and obstructive stressors partially mediated the effect of the use of enterprise social media for social tasks on work exuberance, with the direct effect accounting for 93.83% and the indirect effect accounting for 6.17% of the variation. However, the mediating effect of obstructive stressors on the relationship between the use of enterprise social media and job satisfaction was not significant. Therefore, Hypothesis 6a was supported and Hypothesis 6b was not supported. Overall, the final hypothesis test results of this paper are shown in Table 5.

**TABLE 3:** Mediating effect of the use of enterprise social media for work tasks.

	**Coeff**	**BootSE**	**BootLLCI**	**BootULCI**	
Total effect	0.7438	0.069	0.607	0.88	
Direct effect	0.74	0.0768	0.586	0.8862	99.49
Challenge stressors	0.0039	0.0138	–0.0242	0.032	0.53
Direct effect	0.6982	0.0741	0.5518	0.8438	93.87
Obstructive stressors	0.0456	0.0261	0.001	0.103	6.13

**TABLE 4:** Mediating effect of stressors on social media use.

	**Coeff**	**BootSE**	**BootLLCI**	**BootULCI**	
Total effect	0.7438	0.069	0.607	0.88	
Direct effect	0.6982	0.0741	0.5518	0.8438	93.87
Challenge stressors	0.0456	0.0261	0.001	0.103	6.13
Direct effect	0.1658	0.0394	0.0912	0.2443	89.67
Obstructive stressors	0.0191	0.0131	–0.004	0.0473	11.52

**TABLE 5:** Hypothesis test results.

	**Hypotheses**	**Status**
H1a	The use of enterprise social media for work tasks has a significant positive impact on thriving at work.	Established
H1b	The use of enterprise social media for social tasks has a significant positive impact on thriving at work.	Established
H2a	The use of enterprise social media for work tasks has a significant positive impact on the strength of challenge stressors.	Not established
H2b	The use of enterprise social media for work tasks has a significant negative impact on the strength of obstructive stressors.	Established
H3a	The use of enterprise social media for social tasks has a significant negative impact on challenge stressors.	Established
H3b	The use of enterprise social media for social tasks has a significant negative impact on obstructive stressors.	Not established
H4a	Challenge stressors have a significant negative impact on thriving at work.	Established
H4b	Obstructive stressors have a significant negative impact on thriving at work.	Established
H5a	Challenge stressors mediate the relationship between the use of enterprise social media for work tasks and thriving at work.	Not established
H5b	Obstructive stressors mediate the relationship between the use of enterprise social media for social tasks and thriving at work.	Established
H6a	Challenge stressors mediate the relationship between the use of enterprise social media for work tasks and thriving at work.	Established
H6b	Obstructive stressors mediate the relationship between the use of enterprise social media for social tasks and thriving at work.	Not established

## Discussion

This study profoundly reveals the impact of enterprise social media use on employees. Three aspects are verified.

First, it verifies the impact of social media on employees’ stress perception. Enterprise social media has brought faster information dissemination and instant communication (Al-Oraiqat et al., 2021). According to the theory of the cognitive interaction of stress, individuals’ understanding and evaluations of stress are influenced by external variables (Evan et al., 2014). Therefore, future research on work stress should focus on clarifying the boundary conditions of these effects (Jose et al., 2021). There is no empirical study that considers the use of enterprise social media as a situational variable that affects individuals’ perception of stress, and it is unclear whether enterprise social media use increases or decreases individuals’ perception of stress (Liu and Bakici, 2019). This study confirmed that the use of social media can significantly reduce employees’ challenges and obstructive stressors. This study shows that using social media for work tasks can improve employees’ sense of exuberance by reducing obstructive stressors. To promote this effect, enterprises can establish an open and transparent environment and encourage employees to share knowledge and post information on social media (Ma et al., 2020). To improve employees’ sense of exuberance, enterprises can reduce challenge stressors by creating a stress-free office atmosphere with harmonious interpersonal relationships as the goal (Skade et al., 2020). This will provide a foundation for better cooperation and support in the future.

Second, it expands the research perspective of the impact of social media use on employees’ stress perception and proves that the social function of corporate social media has a positive impact on employees’ stress relief. So far, most researches on enterprise social media have focused on work-related tasks conducted on such media, ignoring other types of enterprise social media usage (Liang et al., 2020). Many enterprises think that using social media at work negatively affects office efficiency and organizational performance, and some organizations even prohibit it. In contrast to this one-sided view, our findings show that social media can alleviate employees’ stress and promote their sense of exuberance. The authors found that both work and social tasks conducted on enterprise social media have a positive impact on work exuberance, but the mechanisms are different. Using enterprise social media for work tasks improves work exuberance by reducing the strength of obstructive stressors, whereas using it for social tasks, which is related to social communication, improves employees’ sense of exuberance by reducing the strength of challenging stressors. Therefore, enterprises should develop social media applications that provide employees with resources, including not only information and knowledge resources for work. Also emotional and networking resources for building social support, which will enhance employees’ sense of control over their work (Leidner et al., 2010).

Third, our findings indicate that the use of enterprise social media helps employees to thrive at work. The authors verified the relationship between social media use and employees’ thriving at work and found that even social media applications with social attributes still have positive impacts on the improvement of employees’ work efficiency. Previous studies have focused on the negative impact of such social attributes (Liang et al., 2020). It is undeniable that they can affect employees’ concentration and interrupt their normal working state (Skade et al., 2020). These social attributes have been seen as having a negative effect on employees’ work efficiency. However, our findings suggest that enterprise social media can provide employees with social support, enhance organizational socialization and the social embeddedness of employees, reduce challenge stressors, and moderate negative emotions caused by heavy workloads or responsibilities. Together, as a key indicator of employees’ sustainable development and physical and mental health, thriving at work occurs when employees have high vitality and are learning new things, as well as having positive impacts on the sustainable development and organizational performance of enterprises. Therefore, both organizations and employees need to take certain measures to enhance employees’ sense of exuberance.

Our research hypotheses that the impact of work-related social media use on challenging stress, the impact of social-related social media use on obstructive stress, and its impact on job exuberance have not been proved. This may occur because work-related social media use provides basic work support rather than support for employees’ further development. For social-related attributes, it is difficult to play a role in the organizational environment with a disharmonious organizational atmosphere (Hase et al., 2018). This can be further studied as an important research direction.

## Limitations and Future Directions

First, at the methodological level, the questionnaire survey method used in this study only collected data at a certain time point and did not test employees’ stress over time. This is a particular limitation for assessing challenge stressors; although challenge stressors consume employees’ psychological resources in the short term (Boswell et al., 2004), in the long term, employees may be compensated for their resource loss. Therefore, in the long term, a challenge stressor may also have a positive impact on employees and enhance their sense of exuberance. In future research, the authors will explore the impact of the use of enterprise social media on thriving at work from a dynamic perspective. Future studies could also include cross-level research that comprehensively considers the team level and organization level factors that affect work prosperity. For example, future research could focus on the influence of team characteristics and organizations’ operations on work prosperity and will improve and enrich the overall theoretical framework.

Second, the samples of this study have limitations. Research shows that social media users with different cultural backgrounds have different usage habits and focus on social media (Huang et al., 2015). The use of corporate social media may have similar impacts (Xu et al., 2019). For example, different cultural backgrounds may make different employees feel different when using social media, and then affect their feelings of stress. However, in this study, all samples are from China, so it is difficult to study the impact of cross-cultural differences on corporate social media applications. At the same time, the applications of social media also have an impact on employees’ work behavior. Different occupations may also have an impact on employees’ social media use (Zhang J. et al., 2015). This study cannot further explore the influence of different occupations on the relationship between social media use and thriving at work due to the limitations of the sample collection. Therefore, whether the findings are restricted to science and technology enterprises or not is worthwhile to study further.

Third, this study does not effectively explore the impact of different regulations and policies and enterprise policies on the use of social media. In addition to culture, laws of different countries, especially privacy protection policies, may also have an impact on the use of different social media (Costello et al., 2016). Under different privacy protection systems, whether employees’ social media use habits are different, and whether it will have an impact on the results of this study. The research on policies and regulations based on this different impact also has very strong practical significance. Overall, the limitations of the research study could provide further research on thriving at work through social media.

## Conclusion

At present, research on thriving at work is still in its infancy. Although there are models in related fields, such as the social embeddedness model and the personal growth integration model, there are obvious gaps in the literature. However, empirical studies are continuously contributing to the development of theoretical frameworks. This paper not only identified new components of the social embeddedness model, including the use of enterprise social media but also explored the mediating role of stressors on personal growth at work.

This study explored the impact of the use of enterprise social media on employees’ thriving at work. The results showed that both social and work-related applications of social media improve employees’ working conditions. In particular, the authors found that social networking applications can play a positive role in reducing employee stress and improving their work status. This contradicts the consensus that using social media for social tasks negatively affects employees’ work and engagement. Accordingly, if the authors expect employees to love their work, the authors should pay attention to the social functions of enterprise social media, and even encourage employees to make full use of these social functions during work hours.

## Data Availability Statement

The original contributions presented in the study are included in the article/supplementary material, further inquiries can be directed to the corresponding author/s.

## Author Contributions

DL: mainly responsible for writing the article. BH: responsible for data analysis. YL: responsible for providing ideas. PL: responsible for topics and resources. All authors contributed to the article and approved the submitted version.

## Conflict of Interest

The authors declare that the research was conducted in the absence of any commercial or financial relationships that could be construed as a potential conflict of interest.

## Publisher’s Note

All claims expressed in this article are solely those of the authors and do not necessarily represent those of their affiliated organizations, or those of the publisher, the editors and the reviewers. Any product that may be evaluated in this article, or claim that may be made by its manufacturer, is not guaranteed or endorsed by the publisher.
